# *Chlamydophila pneumoniae *induces a sustained airway hyperresponsiveness and inflammation in mice

**DOI:** 10.1186/1465-9921-8-83

**Published:** 2007-11-19

**Authors:** Francesco Blasi, Stefano Aliberti, Luigi Allegra, Gioia Piatti, Paolo Tarsia, Jacobus M Ossewaarde, Vivienne Verweij, Frans P Nijkamp, Gert Folkerts

**Affiliations:** 1 Institute of Respiratory Diseases, University of Milan, IRCCS Ospedale Maggiore Fondazione Policlinico-Mangiagalli-Regina Elena, Milano, Italy; 2 Department of Medical Microbiology and Infectious Diseases, Erasmus MC, Rotterdam, The Netherlands; 3 Department of Pharmacology and Pathophysiology, Utrecht Institute for Pharmaceutical Sciences, Utrecht, The Netherlands

## Abstract

**Background:**

It has been reported that *Chlamydophila (C.) pneumoniae *is involved in the initiation and promotion of asthma and chronic obstructive pulmonary diseases (COPD). Surprisingly, the effect of *C. pneumoniae *on airway function has never been investigated.

**Methods:**

In this study, mice were inoculated intranasally with *C. pneumoniae *(strain AR39) on day 0 and experiments were performed on day 2, 7, 14 and 21.

**Results:**

We found that from day 7, *C. pneumoniae *infection causes both a sustained airway hyperresponsiveness and an inflammation. Interferon-γ (IFN-γ) and macrophage inflammatory chemokine-2 (MIP-2) levels in bronchoalveolar lavage (BAL)-fluid were increased on all experimental days with exception of day 7 where MIP-2 concentrations dropped to control levels. In contrast, tumor necrosis factor-α (TNF-α) levels were only increased on day 7. From day 7 to 21 epithelial damage and secretory cell hypertrophy was observed. It is suggested that, the inflammatory cells/mediators, the epithelial damage and secretory cell hypertrophy contribute to initiation of airway hyperresponsiveness.

**Conclusion:**

Our study demonstrates for the first time that *C. pneumoniae *infection can modify bronchial responsiveness. This has clinical implications, since additional changes in airway responsiveness and inflammation-status induced by this bacterium may worsen and/or provoke breathlessness in asthma and COPD.

## Introduction

The association between respiratory infections and asthma exacerbations has been evaluated both for viral agents [1-3], and non-viral respiratory pathogens, such as *Mycoplasma pneumoniae *and *Chlamydophila pneumoniae *[4-8]. Involvement of *C. pneumoniae *in the initiation and promotion of asthma and COPD has been suggested [9-12].

Chlamydiae are obligate intracellular bacteria with a unique growth cycle involving infectious elementary bodies and replicative reticulate bodies [13,14]. Epithelial cells appear to be the primary targets for infection by *C. pneumoniae*, although macrophages are also infected [15,16].

Mice are susceptible to *C. pneumoniae *infections by intranasal inoculation [17] and develop pneumonia with characteristics resembling those of human disease [15,18,19]. *C. pneumoniae *can be isolated from tissues and peripheral blood mononuclear cells, and specific DNA can be detected in the same sites by PCR [20] and by immunohistochemistry [17,21].

The effect of this bacterium on airway responsiveness has not yet been investigated. Inoculation of *M. pneumoniae *in hamsters increases airway hyperresponsiveness to histamine [22], and *M*. *pneumoniae *inoculation in allergen-sensitized mice modulates airway hyperresponsiveness and lung inflammation [23].

*C. pneumoniae *infection in monocytes in vitro induces TNF-α secretion [19] and activates nuclear factor-κB (NF-κB) [24]. The activity of NF-κB is highly correlated to the degree of lung dysfunction and to the course of the disease in an animal model of asthma [25].

A recent multicenter, double-blind, randomized, placebo-controlled clinical study assessed oral telithromycin as a supplement to standard of care treatment for adult patients with acute exacerbations of asthma [26]. Ketolide antibiotic treatment was associated with statistically significant and clinically substantial benefits. In this population 61% of patients had evidence of *C. pneumoniae *and/or *M. pneumoniae *infection and the effect of telithromycin on FEV_1 _was statistically significant in patients with documented infection at baseline and not in those patients without evidence of infection. However, there were no differences between infection-positive and -negative groups in terms of the other study outcomes, so that the mechanisms of benefit remain unclear.

The aim of our study was to evaluate the effect of *C. pneumoniae *infection on airway function in mice and to find possible relations with inflammatory cells and/or mediators and airway pathology, in order to better elucidate the pathophysiologic mechanisms.

## Methods

### Animals

Male BALB/c mice of 5–6 weeks of age were obtained from the Central Animal Laboratory at Utrecht University, The Netherlands. They were housed under controlled condition in macrolon cages containing 8 mice per cage. Water and standard chow were presented ad libitum. Animal care and use were performed in accordance with the guidelines and approval of the Dutch Committee of animal experiments.

### Treatment

Mice were anaesthetized with a short lasting inhalation anesthetic (Halothane) and inoculated intranasally with *C. pneumoniae *strain AR39 in saline (50 μl, 10^6 ^inclusion-forming units (IFU)) at day 0. Tests were performed at days 2, 7, 14 and 21. Control animals were treated in the same way with saline.

### Airway responsiveness in conscious unrestrained mice

Airway responsiveness was measured *in vivo *at day 2, 7, 14 and 21 from the infection using a whole body plethysmograph (Buxco, Sharon, CT, USA) [27]. The plethysmograph consisted of a reference chamber and an animal chamber. The animal chamber was attached to the outside via a pneumotachograph in the top of the plethysmograph. An aerosol inlet to the animal chamber was centrically located in the roof of the animal chamber. When an animal was placed in the animal chamber and was breathing quietly, pressure fluctuated within that chamber. These changes in box pressure represented the difference between tidal volume and thoracic movement during respiration. The differential pressure transducer measured the changes in pressure between the animal chamber and the reference chamber and brings these data to a preamplifier. Thereafter, data were sent to a computer where several parameters were calculated, representing the lung function of the animal. In the present study, mice were exposed for 3 minutes to doubling doses of aerosolized metacholine ranging from 1.56 mg/ml to 25 mg/ml. After exposure to metacholine lung function was measured for 3 minutes. From the known lung function parameters peak expiratory flow (PEF), tidal volume (TV), expiratory time (Te) and frequency (f), the computer calculates the enhanced pause (PenH).

### BAL and differential cell counts

Broncho-alveolar lavage (BAL) was performed in the same animals that were used for *in vivo *airway hyperresponsiveness measurements [27]. Mice were killed by cervical dislocation 2, 7, 14, 21 days after inoculation. The trachea was trimmed free of connective tissue and the upper part was removed for histology (see below). In the lower part of the trachea a cannula was inserted. The lungs were filled with 1 ml aliquots of pyrogen free saline (0.9% NaCl) supplemented with aprotenine in 5% bovine serum albumin of 37°C *in situ*. Fluid was collected in a plastic tube on ice (4°C) (totally 1 ml). This procedure was repeated 3 times with aliquots of pyrogen free saline (0.9% NaCl) and fluid was collected in a separate plastic tube on ice (4°C) and the cell suspensions recovered from each animal were pooled (totally 3 ml). Thereafter, the BAL cells were centrifuged (400 *g*, 4°C, 5 min) and the supernatant from the 1 ml aliquots were collected and stored at -30°C till IFNγ, MIP-2 and TNF-α were measured by ELISA. The pellets from the 1 ml and 3 ml aliquots were pooled and re-suspended in totally 150 μl PBS (4°C). The total number of BAL cells was counted by use of a Bürker-Türk chamber. For differential BAL cell counts cytospin preparations were made and stained with Diff-Quick (Merz & Dade A.G., Düdingen, Switzerland). Cells were differentiated into macrophages, lymphocytes, neutrophils and eosinophils by standard morphology. At least 200 cells per cytospin preparation were counted and the absolute number of each cell type was calculated.

### INF-γ, MIP-2, and TNF-α ELISA

INF-γ, MIP-2, and TNF-α were analysed as previously reported [28-30]. Flat-bottom microplates (96-wells, Maxisorp, Nunc, Life Technologies, Breda, The Netherlands) were coated for over night at 4°C with capture antibody (100 μl per well) purified Rt α Ms IFNγ, purified Rt α Ms MIP-2, purified Rt α Ms TNF-α (BioSource International, Inc., Camarillo, USA). After coating, plates were washed with PBS containing 0.05% Tween-20, and blocked with ELISA-buffer (2 mM EDTA, 136.9 mM NaCl, 50 mM Tris, 0.5% BSA and 0.05% Tween-20, pH 7.2) at room temperature (RT) for 1 hour while gently shaking. After removing the ELISA buffer, 100 μl of samples and standards (rmIFNγ, rmMIP-2, or rmTNF-α (BioSource) were applied and incubation was continued at RT for 2 hours. Thereafter, the second antibody diluted in ELISA-buffer was added followed by incubation at RT for 2 hours while shaking. After washing, 100 μl anti-DIG-POD (anti-Digoxigenin conjugated with horse-radish peroxidase) (Roche Diagnostics) was applied and incubation was continued at RT for 1 hour. After washing, streptavidin-peroxidase (0.1 μg/ml, CLB) was added and incubation was performed at RT for 1 hour. After washing the plates, 0.4 mg/ml o-phenylenediamine-dihydrochloride in PBS containing 0.04% hydrogen peroxide was added. After approximately 5 minutes the reaction was stopped by adding 4 M H_2_SO_4_. Subsequently, optical density was measured at 492 nm.

### Preparation of specimens for scanning electron microscopy observation

At day 2, 7, 14, 21 tracheas were removed, gently washed in 0.9% saline solution and immediately fixed in 4% formaldehyde fixative [31]. After fixation, they were opened longitudinally and dehydrated in increasing alcohol series. A Critical Point drying (Balzers CPD 030) was performed and finally specimens were mounted on aluminum stubs with carbon double-sided adhesive tape and sputter-coated with 200 Angstrom of gold (Baltec SCD 005). Samples were examined under scanning electron microscopy (Philips 505).

### Statistical analysis

Data are represented as mean (± standard error [SEM]). Differences between groups were compared using an unpaired, two-tailed Student's t-test. A p value < 0.05 was considered significant. Each group consists of ≥7 animals.

## Results

### Airway responsiveness

The *in vivo *airway responsiveness following increasing concentrations of aerosolized methacholine in spontaneously breathing mice was measured by using a barometric plethysmograph (PenH).

**Figure 1:**
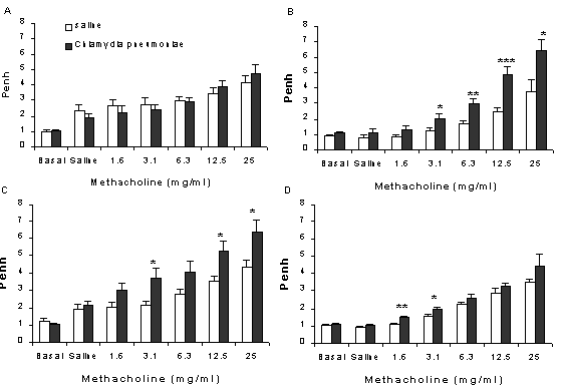
Airway responsiveness to increasing concentrations of methacholine at various points after inoculation of mice with saline (open bars) or *C. pneumoniae *(black bars). A: Day 2; B: Day 7; C: Day 14, D: Day 21. (*p < 0.05; **p < 0.001; ***p < 0.005, n = 7–8). Unrestrained plethysmograph measurements were performed for 3 min after each exposure to methacholine and expressed as Penh-values.

Basal PenH values did not differ between the experimental groups (Day 2–21, Fig 1). At day 2, exposure to saline nebulization slightly increased PenH in both experimental groups (Fig 1A). Moreover, metacholine concentration-dependently increased PenH and, again, there was no difference between saline- and *C. pneumoniae*-treated animals. Interestingly, on day 7 airway responsiveness was significantly increased in the *C. pneumoniae *– compared to the saline-treated group. At every concentration of metacholine, the PenH was almost doubled (Fig 1B). Similar results were obtained on day 14 (Fig 1C). On day 21 the airway hyperresponsiveness in *C. pneumoniae*-treated animals fainted and significant changes were only observed at lower concentration of methacholine (Fig 1D). These data indicate that *C. pneumoniae *infection induces a sustained airway hyperresponsiveness.

### Airway inflammation

To assess whether *C. pneumoniae *infection induces a change of the inflammatory cell numbers in the lungs, the total number of cells and the absolute number of macrophages, neutrophils and lymphocytes were counted in the bronchoalveolar lavage fluid. There were no eosinophils in the BAL-fluid of the experimental groups (Day 2–21).

**Figure 2:**
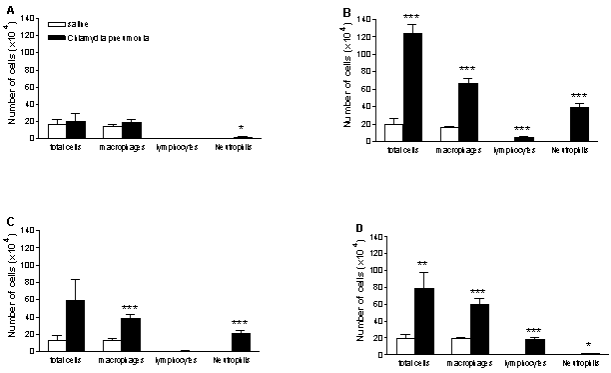
Number of bronchoalveolar cells obtained by lung lavage at various points after inoculation of mice with saline (open bars) or *C. pneumoniae *(black bars). A: Day 2; B: Day 7; C: Day 14, D: Day 21. (*p < 0.05; **p < 0.005; ***p < 0.0001, n = 7–8).

Two days after the inoculation there was no difference between the experimental groups with respect to total cell numbers, however, there was a slight but significant increase in the number of neutrophils in the *C. pneumoniae*-group (Fig 2A). There was a prominent inflammation on day 7 and all the different cell types were increased in the *C. pneumoniae-*group (Fig 2B). The inflammation was slightly less 14 days after infection but still a significant increase in macrophages and neutrophils was observed in the *C. pneumoniae*-group (Fig. 2C). Comparable results were obtained on day 21, however now there was a significant increase in the number of lymphocytes and the increase in neutrophils was comparable with day 2 (Fig 2A &2D).

### INF-γ, MIP-2, and TNF-α in BAL

**Figure 3:**
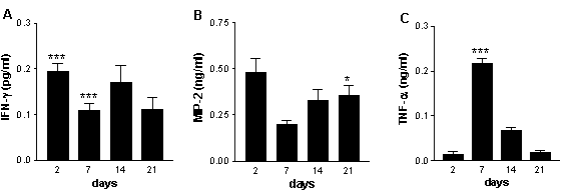
Concentrations of: **A **IFN-γ (pg/ml) **B **MIP-2 (pg/ml) **C **TNF-α (pg/ml) in the bronchoalveolar lavage fluid 2, 7, 14, and 21 days after *C. pneumoniae *infection of mice. Data are presented as mean ± SEM, n = 7–8. P < 0.005 ***, p < 0.0001 compared to the saline groups. #p < 0.01 compared to the *C. pneumoniae *group on day 2.

Several cytokines were measured in the BAL fluid to find a possible relation between activation and influx of cells. Since basal levels of cytokines did not differ between the experimental days, the saline treated groups were pooled. In *C. pneumoniae*-treated animals, the IFN-γ levels significantly increased to more than 100 pg/ml throughout the study (Fig 3A). On day 2, 14 & 21 MIP-2 concentrations were 40% enhanced in *C. pneumoniae*-treated animals compared to the control group. On day 7 however, MIP-2 levels dropped to control levels and were significantly decreased compared with day 2 (Fig 3B).

Interestingly, TNF-α was increased on day 7 (compared both to the control group and day 2 after *C. pneumoniae*), after which the concentrations dropped to control levels on day 14 and 21 (Fig. 3C).

### Scanning electron microscopy

**Figure 4:**
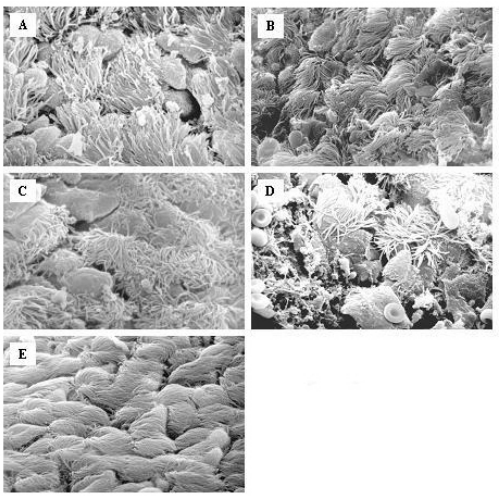
Scanning electron microscopy of the epithelial layer of the trachea from mice inoculated saline (**E**) or at various points after infection with *C. pneumoniae*: **A**: Day 2; **B**: Day 7; **C**: Day 14, **D**: Day 21 (2000–3000×). Two days after infection, the respiratory epithelium showed hyperthrophy of goblet cells and some scattered bacteria that were observed prevalently in contact with ciliated cells (Fig. 4A). After 7 days ciliary disorientation was the most evident change and there were a few single chlamydial bodies (Fig. 4B). The epithelium appeared severely damaged on day 14, goblet cells were notably hypertrophic and numerous bacteria were visible, also in little microcolonies (Fig. 4C). In addition to exfoliated epithelial cells on day 21 (Fig 4D), in some areas shorter cilia began to appear, a marker of ciliary regeneration.

All samples obtained from saline-treated animals showed no alterations of ciliated or secretory cells (Fig. 4E). In contrast, the respiratory epithelium of mice infected with *C. pneumoniae *after 2 days showed hyperthrophic goblet cells and some scattered bacteria that were observed predominantly in contact with ciliated cells (Fig. 4A). After 7 days ciliary disorientation was the most evident change, hyperplasia and hypertrophia of the secretory cells were noticeable and there were a few single chlamydial bodies (Fig. 4B). Detached cells were observed only occasionally. The most relevant alterations were seen 14 days after infection: the epithelium appeared severely damaged, goblet cells were notably hypertrophic and numerous bacteria were visible, also in little micro-colonies (Fig. 4C). After 21 days a mucus component was present and the normal architecture of the respiratory epithelium was lost (Fig. 4D); in addition to exfoliated epithelial cells, in some areas shorter cilia began to appear, a marker of ciliary regeneration.

## Conclusion

*C. pneumoniae *infection may be a cofactor in the pathogenesis of airway diseases such as asthma and COPD [11,32-34]. It has been suggested that acute infection with *C. pneumoniae *is associated with new onset of asthma [4,10], and *C. pneumoniae *and *M. pneumoniae *infections are involved in acute exacerbations of asthma. Data on chronic *C. pneumoniae *infection in COPD patients indicate this agent as a plausible candidate for the modulation of the natural history of chronic bronchitis and emphysema [12,35-37].

Atypical pathogen persistent infection may participate in airway inflammation. Chlamydial infection activates a cytokine response including basic fibroblast growth factor [38] by smooth muscle cells, and TNF-α secretion by monocytes [39]. TNF-α production is induced by *C. pneumoniae *heat shock protein 60 (HSP60) [40] and is associated with neutrophil influx and endothelial and epithelial expression of IL-1 and adhesion molecules [41,42]. HSP60 also induces matrix metalloproteinases (MMPs) production by macrophages, particularly of MMP-9, an enzyme are felt to be involved in the pathogenesis of emphysema [42]. Moreover, an association was observed between the anti-*C. pneumoniae *heat shock protein 10 antibodies and adult onset asthma [34].

However, no data have so far been obtained in demonstrating a role for *C. pneumoniae *infection in the pathogenesis of airway hyperresponsiveness in vivo. In our study we evaluated the effect of acute *C. pneumoniae *infection on bronchial reactivity in mice. This model allowed the direct evaluation over time of the effects of the infection on epithelial damage, cellular influx and cytokines in the airways in relation to bronchial response to metacholine challenge. Based on the results obtained, a likely sequence of events can be proposed. The inoculation of *C. pneumoniae *into the respiratory tract may trigger alveolar macrophages to produce IFN-γ and MIP-2. Both cytokines are increased in the BAL-fluid as early as two days after inoculation and attract and activate immune cells in order to eliminate the infection with the bacteria. The production of MIP-2 on day 2 might explain the slight but significant neutrophil influx. At the same time, *C. pneumoniae*, actively infects cells with the goal of endocellular replication. Epithelial cells appear to be the primary targets, although other studies have shown that macrophages are also infected [21]. On the basis of scanning electron microscopy findings, we observed that inoculation of *C. pneumoniae *resulted in epithelial damage and secretory cell hypertrophia. These lesions were present in the early phase post-inoculation (day 2) and this might be explained by bacterial penetration into the epithelial cells and by mediators (such as reactive oxygen species) that are released by macrophages during elimination of *C. pneumoniae *[43]. Evidence of *C. pneumoniae *replication was found on day 7. Similar results were obtained in a recent study, in which replication *C. pneumoniae *was measured in supernatants of individual lung suspensions of mice in time and peaked at day 7 [17]. In addition to the presence of chlamydial bodies in the epithelial layer (Fig 4B), the inflammatory cell influx and the level of TNF-α peaked on this day. It is likely that the increase in TNF-α contributes to the huge neutrophil influx at this time point since this cytokine is a potent chemoattractant and activator for neutrophils. The obvious increase of TNF-α on day 7 might be due to the release of *C. pneumoniae *that replicated in the epithelial cells. In contrast to what was seen with TNF-α, MIP-2 levels dropped significantly compared with day 2, and increased again on day 14 and 21. At the latter two time points, MIP-2 might be responsible for the (less pronounced) increase in neutrophils, since TNF-α was hardly present. The reason for the hypobolic synthesis pattern for MIP-2 is unclear.

The sustained increase in INF-γ is probably due to the particular life cycle of this bacterium. Following completion of the replication stage, the reticulate bodies once again mature into elementary bodies that are released after lyses of the infected cell and may infect other cells. It is likely that the afore mentioned process and the release of the inflammatory mediators are responsible for the epithelial damage observed up until day 21. Epithelial damage increased over time and was associated with airway hyperresponsiveness. However, when evidence of cellular regeneration was observed (day 21), this coincided with a drop in the degree of hyperresponsiveness. This suggests that epithelial damage following *C. pneumoniae *inoculation may at least partly be responsible for alterations in airway responsiveness [43]. It is not likely that the airway hyperresponsiveness is due to a *C. pneumoniae*-induced change in histamine synthesis [17] or metabolism [22], since the mice were exposed to a cholinergic agonist.

A further finding was that acute infection is followed by a striking increase of cellular influx after 7 days that persisted till day 21. Neutrophil influx starts at day 2, reaching the peak at day 7 with a four-fold increase in the number of macrophages. It has to be stressed that there was no influx of eosinophils. Crimi *et al*., [44] suggested, that the degree of hyperresponsiveness in asthma patients may be correlated with factors other than eosinophil inflammation. One of these additional factors could be the immune- and inflammatory-mediators released by other cells.

In summary, our study provides the first evidence that *C. pneumoniae *infection can modify bronchial responsiveness in mice. The induction of the airway hyperresponsiveness might be due to inflammation and morphological changes of the epithelial layer. These changes could be induced by the infection itself and by the mediators released by the inflammatory cells (such as cytokines and reactive oxygen species). The future challenge is to substantiate the clinical significance of these results by investigating 1) *C. pneumoniae *infection in animal models for asthma and COPD (i.e. ovalbumin sensitized and challenged mice and mice exposed to cigarette smoke, respectively) and 2) anti-microbial therapy in (subgroups) of asthma- [16,26] and COPD- [45] patients.

## Competing interests

F. Blasi, S. Aliberti, L. Allegra, G. Piatti, P. Tarsia, JM Ossewade, V. Verweij, FP Nijkamp, and G Folkerts, all have no personal financial support or are involved in organizations with financial interest in the subject matter, and present no actual or potential competing interests.

## Authors' contributions

FB conceived the study, participated in its design, coordination and drafted the manuscript, SA participated to the design of the study and to electron microscopy studies, LA participated in the study design and coordination, GP performed scanning electron microscopy, PT participated in the study design and in drafting the manuscript, JMO participated in the animal studies and supplied *Chlamydophila pneumoniae *strains, VV participated in the animal studies, FPN participated in the study design and coordination, GF conceived the study, participated in its design, coordination and drafted the manuscript.

All the authors read and approved the final manuscript.
